# Characterization of the complete chloroplast genome of the *Solanum tuberosum* L. cv. Favorita (Solanaceae)

**DOI:** 10.1080/23802359.2021.1886885

**Published:** 2021-03-16

**Authors:** Shuang Li, Ya-Ping Wang, Yan-Fei Zhao, Jia-Yue Zhang, Jing-Ying Zhang, Hao-Ran Ma, Yue Yue, Chun-Yang Du, Chun-Bo Zhao, Yu-Zhu Han

**Affiliations:** a College of Horticulture, Jilin Agricultural University, Changchun, P.R. China; b Management Office of Teaching and Scientific Research Base, Jilin Agricultural University, Changchun, P.R. China; c College of Resource and Environmental, Jilin Agricultural University, Changchun, P.R. China

**Keywords:** *Solanum tuberosum*, complete chloroplast genome, phylogenetic analysis, Solanaceae

## Abstract

Potato (*Solanum tuberosum* L.), a species of the family Solanaceae, is the fourth most important food crop worldwide. *Solanum tuberosum* L. cv. Favorita is a long oval, smooth, yellowish-skinned potato variety with green and plump leaves. It has a dry matter content of 17.7% and starch content of 12.4–14.01% in the tuber. In order to support more genetic data for the taxonomy of *S. tuberosum*, the complete chloroplast (cp) genome sequence of *S. tuberosum* L. cv. Favorita was determined using next-generation sequencing. In leaves, the chloroplast genome accounts for 5.17% of the total genome. The entire cp genome was determined to be 155,296 bp in length. It contained large single-copy (LSC) and small single-copy (SSC) regions of 85,737 and 18,373 bp, respectively, which were separated by a pair of 25,593 bp inverted repeat (IR) regions. The genome contained 132 total genes, including 87 protein-coding genes, 37 tRNA genes, and eight rRNA genes. The overall GC content of the genome is 37.9%. A phylogenetic tree reconstructed by 60 chloroplast genomes reveals that *S. tuberosum* L. cv. Favorita is most closely related to *S. tuberosum* L. cv. Desiree and *S. tuberosum* L. cv. Atlantic.

*Solanum tuberosum* L. (family: Solanaceae) has high nutritional value, adaptability, and large yield. It is the largest non-cereal food crop worldwide and ranked as the world's fourth most important food crop after rice, wheat, and maize. (Horton and Sawyer 1985; Zhang et al. 2017). *Solanum tuberosum* L. cv. Favorita (https://www.europotato.org/varieties/view/Favorita-E#/) is a long oval, smooth, yellowish-skinned potato variety with green and plump leaves. Compared with other varieties, its tubers have a medium dry matter content of 17.7% and starch content of 12.4–14.01%. Since the main sites of starch synthesis are amyloid and chloroplast, and the chloroplast genome contains many genes involved in starch synthesis, it is necessary to characterize the chloroplast genome of the potato Favorita. In addition, because Favorita is an important parent material for potato breeding, the chloroplast genome will enrich the genetic information for potato Favorita sprout mutation breeding and cross-breeding (Duan et al. 2019).

Healthy leaf samples were collected from a tissue culture plant (E:125.417353, N43.821995). The total genomic DNA was extracted from the fresh leaves of *S. tuberosum* L. cv. Favorita using the DNeasy Plant Mini Kit (Qiagen, Valencia, CA, USA). The voucher specimen (JAUHL07) was deposited at the Herbarium of College of Vegetable Science, Jilin Agricultural University. After DNA isolation, 1 μg of purified DNA was fragmented and used to construct short-insert libraries (insert size ∼350 bp) according to the manufacturer’s instructions (BGISEQ) detailed in the previous literature (Huang et al. 2017). Then DNA libraries were sequenced by Hefei Bio&Data Biotechnologies Inc. (Hefei, China) on the BGISEQ-500 platform with PE150 read lengths. The filtered reads were assembled using the program NOVOPlasty Version 3.8.3 (Dierckxsens et al. 2017). The cp-genome was annotated with the GeSeq (Tillich et al. 2017) and tRNAscan (Schattner et al. 2005).

In leaves of *S. tuberosum* L. cv. Favorita, the chloroplast genome accounts for 5.17% of the total genome. Such a high proportion of the chloroplast genome may have contributed to its green and plump leaves and high starch production. The chloroplast genome was determined to comprise double stranded, circular DNA of 155,296 bp containing two inverted repeat (IR) regions of 25,593 bp each, separated by large single-copy (LSC) and small single-copy (SSC) regions of 85,737 and 18,373 bp, respectively (Genbank acc. no. MW307948). The genome contained 132 total genes, including 87 protein-coding genes, 37 tRNA genes, and eight rRNA genes. Seven protein-coding genes, six tRNA genes and four rRNA genes were duplicated in IR regions. Nineteen genes contained two exons and four genes (clpP and ycf3 and two rps12) contained three exons. The overall GC content of *S. tuberosum* L. cv. Favorita cp genome is 37.9% and the corresponding values in LSC, SSC and IR regions are 36.0, 32.1 and 43.1%, respectively. Heteroplasmy testing showed that there are about 134 low-frequency SNP sites with minor allele frequency (MAF) ≥0.03 and ≥5× reads coverage in the chloroplast genome of potato Favorita. Most of these SNPs are located between ycf15 and trnL-CAA.

To investigate its taxonomic status, a maximum-likelihood (ML) was reconstructed based on whole chloroplast genomes from 59 *Solanum* species and one outgroup species (*Eucommia ulmoides*) by Mafft version 1.4 and FastTree version 2.1.10 (Price 2010). The ML phylogenetic tree shows that *S. tuberosum* L. cv. Favorita is most closely related to *S. tuberosum* L. cv. Desiree and *S. tuberosum* L. cv. Atlantic. with bootstrap support values of 100% (Figure 1). Chloroplast genome of *S. tuberosum* L. cv. Favorita adds valuable information for understanding the phylogenetic position of *S. tuberosum* in the genus *Solanum*.

**Figure 1.**
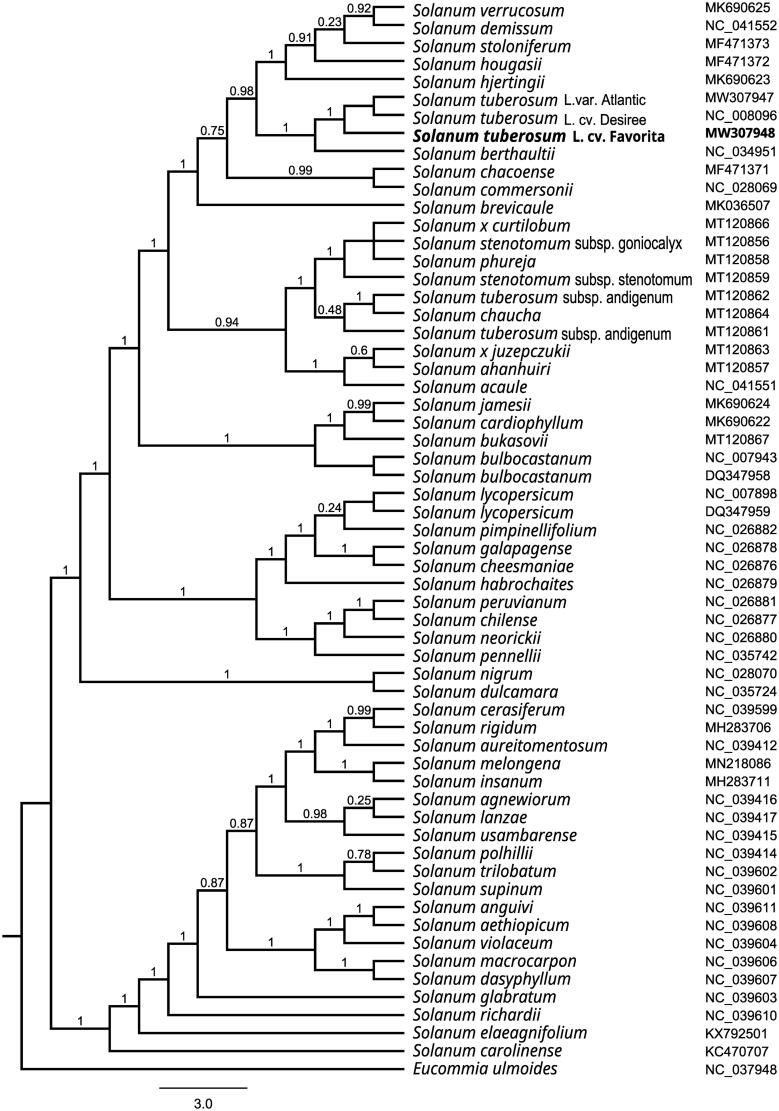
Maximum-likelihood phylogenetic tree based on whole chloroplast genomes from 59 *Solanum* species and one outgroup species (*Eucommia ulmoides*) and the support values are shown at the branches.

## Data Availability

The data that support the findings of this study are openly available in NCBI at Genbank with accession number MW307948 (https://www.ncbi.nlm.nih.gov/nuccore/MW307948.1). Raw sequencing reads used in this study was deposited in the public repository SRA with accession number SRR13162919 (https://www.ncbi.nlm.nih.gov/sra/?term=SRR13162919).

## References

[CIT0001] Dierckxsens N, Mardulyn P, Smits G. 2017. NOVOPlasty: de novo assembly of organelle genomes from whole genome data. Nucleic Acids Res. 45(4):e18–e18. 28204566 10.1093/nar/gkw955 PMC5389512

[CIT0002] Duan Y, Liu J, Xu J, Bian C, Duan S, Pang W, Hu J, Li G, Jin L. 2019. DNA fingerprinting and genetic diversity analysis with simple sequence repeat markers of 217 potato cultivars (*Solanum tuberosum* L.) in China. Am J Potato Res. 96(1):21–32.

[CIT0003] Huang J, Liang X, Xuan Y, Geng C, Li Y, Lu H, Qu S, Mei X, Chen H, Yu T, et al. 2017. A reference human genome dataset of the BGISEQ-500 sequencer. Gigascience. 6(5):1–9. 10.1093/gigascience/gix024 PMC5467036 28379488

[CIT0004] Horton D, Sawyer RL. 1985. The potato as a world food crop, with special reference to developing areas. Potato Physiol. 1–34.

[CIT0005] Price MN, Dehal PS, Arkin AP. 2010. FastTree 2-approximately maximum-likelihood trees for large alignments. PLoS One. 5(3):e9490]. 20224823 10.1371/journal.pone.0009490 PMC2835736

[CIT0006] Schattner P, Brooks AN, Lowe TM. 2005. The tRNAscan-SE, snoscan and snoGPS web servers for the detection of tRNAs and snoRNAs. Nucleic Acids Res. 33(Web Server issue):W686–W689. 15980563 10.1093/nar/gki366 PMC1160127

[CIT0007] Tillich M, Lehwark P, Pellizzer T, Ulbricht-Jones ES, Fischer A, Bock R, Greiner S. 2017. GeSeq - versatile and accurate annotation of organelle genomes. Nucleic Acids Res. 45(W1):W6–W11. 28486635 10.1093/nar/gkx391 PMC5570176

[CIT0008] Zhang H, Xu F, Wu Y, Hu H-h, Dai X-f. 2017. Progress of potato staple food research and industry development in China. J Integr Agric. 16(12):2924–2932.

